# Influence of Genotypic and Environmental Factors on Tobacco Leaves Based on Metabolomics

**DOI:** 10.3390/life12040590

**Published:** 2022-04-15

**Authors:** Bo Fu, Junyang Liang, Mengmeng Zhang, Mingqin Zhao, Xiaoming Ji, Jing Wang

**Affiliations:** College of Tobacco Science, Henan Agricultural University, 63 Nongye Road, Zhengzhou 450002, China; fubo@henau.edu.cn (B.F.); liangjunyang@henau.edu.cn (J.L.); zhangmeng@henau.edu.cn (M.Z.); zhaomingqin@henau.edu.cn (M.Z.); xiaomingji@henau.edu.cn (X.J.)

**Keywords:** ecological functions, metabolites, planting regions, biomarker metabolites, environment

## Abstract

Environmental factors affect plant metabolites, different climates, cultivation conditions, and biotic stresses and genotypes strongly affect their chemical composition and contents. Our aim is to examine the environmental and genetic interaction effects on tobacco metabolite composition. UPLC-QTOF MS/MS coupled with multivariate data analyses were applied for the metabolomics analysis of three tobacco cultivars from different planting regions in China. Principal component analysis (PCA) revealed that environmental factors have a greater effect on tobacco metabolism compared to genotypes. Twelve biomarkers were screened by orthogonal partial least squares discrimination analysis (OPLS-DA). Univariate analysis indicated that Malate, conjugated chlorogenic acid, chlorogenic acid, quercetin 3-rutinoside-7-glucoside, and unknown compound **5** were only influenced by environmental factors (independent of genotype). Quinate, neochlorogenic acid, and ouabagenin, taxezopidine K1, taxezopidine K2, and taxezopidine K3 in tobacco were influenced by the interaction of environmental factors and the genotype. Our results suggest that metabolomics based on UPLC-QTOF MS/MS could be used to analyze the ecological functions of biomarker metabolites and understand the mechanisms of plant adaption to the environment.

## 1. Introduction

Tobacco (*Nicotiana tabacum*) is an ideal model plant for molecular ecology studies, and the metabolites of tobacco are susceptible to environmental and genetic changes. As previously stated, different climates, cultivation conditions, biotic stresses and genotypes strongly affect their chemical composition and contents [1]. Solanaceae is one of the most studied plant families. The most notable active natural products found in these plants are alkaloids, physalins, and withanolides [2]. The Solanaceae family produces a range of biologically active alkaloids, including nicotine and tropane alkaloids. Increasing the ornithine decarboxylase activity in *N. rustica* can increase nicotine production. Overexpression of tobacco PMT (putrescine N-methyltransferase) cDNA increased the nicotine content in *N. sylvestris*. The suppression of endogenous PMT activity severely decreased the nicotine content [3,4].

The contents of volatiles, nicotine and pigments in tobacco have been shown to vary with different cultivars and cultivation conditions [5,6]. In addition, environmental factors, such as drought, low temperature and UV-B radiation, have been reported to affect tobacco metabolites [7]. The interaction influences of genotypes and environmental factors on plant metabolites have been widely studied [8]. However, previous research has mainly focused on specific analyte classes. With the technical advances of metabolomics, research on the metabolic changes of the entire plant by internal and external factors has become fashionable [9]. The utilization of UPLC-QTOF MS/MS is one of the key technologies for metabolomics, which can qualify and quantify relatively complicated secondary metabolites with accuracy. For secondary metabolites, the response to the environment, genotype or systemic interaction can be monitored through metabolomics with LC-MS [10], which is helpful for the comprehensive understanding of the ecological and physiological functions of secondary metabolites and their mechanisms regarding plant metabolism.

In this experiment, we analyzed the tobacco leaves from Xiangxian, Henan Province and Yanji, Jilin Province in China, to determine the influence of the genotype, environment and their interactions on plant metabolites using UPLC-QTOF MS/MS. The ecological and physiological roles of biomarker metabolites were further studied to better understand the mechanisms of plant adaption to the environment. 

## 2. Materials and Methods

### 2.1. Plant Material, Growth Conditions and Experimental Design

The tobacco leaves were collected from two cultivation locations in China: Xiangxian (XX, 33.82° N, 113.44° E) and Yanji (YJ, 42.75° N, 129.39° E) at 9:00 AM on the 5, 15, and 25 August 2015. Cultivars in XX were from ‘Yunyan 87’ (XXY) and ‘Zhongyan 100’ (XXZ), and cultivars in YJ were from ‘Yunyan 87’ (YJY) and ‘Jiyan NO.9’ (YJJ). Each sample was analyzed with five replicates, and every replicate was blended from three different middle leaves. The leaves were flash-frozen in liquid nitrogen and stored at −80 °C until ready for processing. Detailed sample information is provided in Table 1. The information regarding average temperature, total sun exposure time, average humidity, and total rainfall from May to August 2015 in Jilin and Henan were obtained from the China Meteorological Administration (http://www.cma.gov.cn/) (accessed on 31 August 2015).

### 2.2. Sample Extraction

The samples were ground into powder after freeze-drying in liquid nitrogen. A total of 1.5 mL of methanol (75%) with 0.1% formic acid was added to the powder (approximately 20 ± 0.1 mg) for the extraction. The extracted solution was subsequently sonicated for 15 min. After 10 min of centrifugation (12000 r min^−1^) at room temperature, the supernatant was transferred into a microtube and filtered through a 0.22 μm PTFE membrane (Waters).

### 2.3. Soil Composition of the Selected Regions

The basic physicochemical properties of the soil at 0–20 cm depth were as follows: 

Xiangxian: organic matter (SOM) 19.09 g·kg^−1^, available nitrogen (AN) 74.71 mg·kg^−1^, available phosphorus (AP) 8.72 mg·kg^−1^, available potassium (AK) 114.5 mg·kg^−1^, pH 7.8.

Yanji: SOM 24.87 g·kg^−1^, AN 159.25 mg·kg^−1^, AP 29.13 mg·kg^−1^, AK 142.65 mg·kg^−1^, pH 6.5.

### 2.4. LC-MS/MS

Two microliters of the extraction solution were used for analysis by the UPLCQ-TOF MS system (Waters Corporation, Milford, MA, USA) in conjunction with an Acquity BEH C18 column (2.1 mm × 100 mm and 1.7 μm). The analysis sequence of the different samples was randomised. Mobile phase A: ultrapure water with formic acid (0.1%); mobile phase B: acetonitrile with formic acid (0.1%). A gradient elution system was used: 95% A from 0 to 2 min, 95–25% A for 2–24 min, 25% A for 24–26 min, and 25–95% A for 26–28 min, and finally, 95% A for 28–30 min; this system was used as a rinse, and the column flow rate was 400 μL min^−1^. The mass spectrometry detector was equipped with an ESI ion source, and ionisation was performed in the negative (ESI^−^) mode. The ESI ion source parameters were as follows: scan range, 100–1000 *m*/*z*; capillary voltage, 2.5 kV; sample cone voltage, 21 V; collision energy, 15–60 V; source temperature, 100 °C; and desolation temperature, 350 °C. The cone and desolation gas flow was 50 L h^−1^ and 700 L h^−1^, respectively. All of the analyses were acquired using Lockspray to ensure accuracy. Leucine enkephalin (556.3 ng mL^−1^) was used as the lockmass, and the flow rate was 0.4 mL min^−1^. 

### 2.5. Data Analyses

Data preprocessing, including alignment, peak detection, and peak integration and retention time (RT) correction, was performed using Markerlynx XS™ software (Waters Corporation, Milford, CT, USA). The optimized parameters are an RT range of 1–24 min, a mass range of 100–1000 Da, a mass tolerance of 0.02 D, and an RT window of 0.2 min. The data were normalized to the total intensity (area) using Markerlynx. The preprocessed data were then imported into SIMCA-P version 12.0 (version 12.0, Umetrics, Umea, Sweden) for principal component analysis (PCA) and orthogonal partial least squares discrimination analysis (OPLS-DA). The data were scaled with Pareto scaling. Kruskal-Wallis ANOVA and multiple comparisons were conducted using MATLAB software. The potential biomarkers were extracted from S-plots constructed by OPLS-DA [11], which can satisfy the condition when the VIP value is greater than 1, and Kruskal-Wallis ANOVA was simultaneously determined at the level of *p* < 0.01.

For metabolite identification, the elemental composition of the unknown compounds was deduced with the accurate mass to charge ratio (*m*/*z*) and isotopic abundance pattern by the Marker lynx software [12]. Using the molecular formula and the accurate *m*/*z*, a search for the possible chemical structures was conducted in the METLIN database (http://metlin.scripps.edu/) (accessed on 1 January 2016), Chemspider (http://www.chemspider.com/), (accessed on 1 January 2016), KEGG (http://www.genome.jp/kegg/), (accessed on 1 January 2016), Massbank (http://www.massbank.jp/) (accessed on 1 January 2016), and PubChem (http://pubchem.ncbi.nlm.nih.gov/) (accessed on 1 January 2016), Through MS/MS analysis of the unknown peaks by LC-QTOF MS/MS, high-resolution fragmentation information was obtained. Then, the potential chemical structures were screened with MS/MS data to determine the possible chemical structures. 

## 3. Results and Discussion

### 3.1. PCA Analysis of UPLC Q-TOF/MS Data

**Figure 1:**
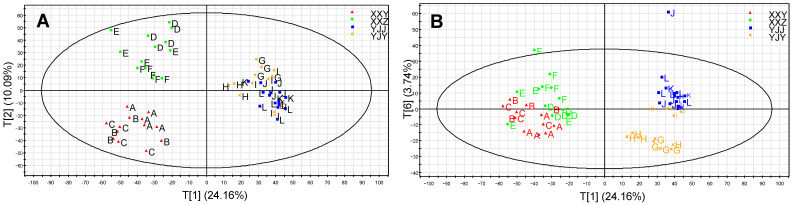
The PCA score plot is marked with different planting regions and cultivars. (**A**) PCA plots with the scores of the first two principal components, and (**B**) PCA plots with the scores of the first principal component and a sixth principal component. (▲) The group in the XXY samples; (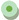) the group in the XXZ samples; (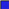) the group in the YJJ samples; (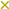) the group in the YJY samples.

Metabolic profiling of the tobacco leaves was acquired by a UPLC-QTOF MS system in the negative ion modal (ESI^−^), and 2489 peaks were detected. The PCA was used to obtain a preliminary overview of the similarities and differences among the samples (Figure 1). Six principal components (PC) were retained in the final PCA model (R^2^X = 0.576, Q^2^ = 0.297). Tobacco samples from different planting regions were well separated by PC1. However, plant metabolic regulation was influenced by both environmental factors and genotypes. Samples of two cultivars grown in XX were separated by PC2, whereas two cultivars grown in YJ were separated based on PC6 (Figure 1B). PCA analysis showed that the influence of the planting regions on tobacco metabolism was greater than the cultivars. In addition, the growth periods had minimal impact on the metabolites compared with the planting regions and cultivars. The cultivars (XXY and XXZ) in XX were discrepant in their metabolite levels in different growth periods. Meanwhile, samples G and H from YJY were significantly different from sample I according to PC6, as the sample I clustered with samples J, K and L, but was distant from samples G and H. However, there was no visible difference between the samples of YJJ in the different periods. Since the temperature in YJ was low in early August and increased significantly in late August, this indicated that the cultivar of Yunyan 87 was more susceptible to environmental change than Jiyan 9. The differences in the climatic conditions might be the main factor causing different metabolite levels in different planting regions and growth periods. 

### 3.2. OPLS-DA Analysis of UPLC Q-TOF/MS Data

**Figure 2:**
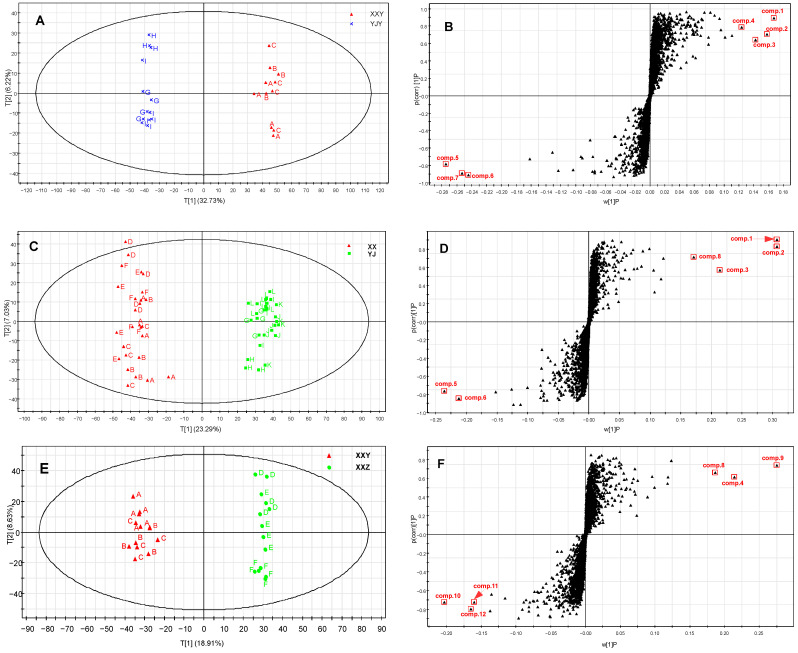
The score plot (**A**,**C**,**E**) and S-plot of OPLS 1 and 2 (**B**,**D**,**F**) generated from the OPLS model. (**A**,**C**,**E**): the OPLS DA score plot of tobacco leaves of different planting regions and different cultivars; (**B**,**D**,**F**): The biomarkers were screened by S-plot based on different planting regions and different cultivars.

Yunyan 87 samples from different planting regions (A, B, C and G, H, I) were separated by the PCA analysis, which demonstrated that the metabolites of tobacco leaves from two planting regions had significant differences. It was more obvious to discriminate between these two sample groups by the OPLS-DA method. Two significant components described 99% of the variation in Y (R^2^Y = 0.994) and predicted 96% (Q^2^Y= 0.956) according to the cross-validation. Seven different metabolites from two planting regions were selected as potential biomarkers by OPLS-DA (Figure 2A,B). Furthermore, comp. **1**, comp. **2**, comp. **3** and comp. **4** were increased in the YJY group, whereas comp. **5**, comp. **6**, and comp. **7** were increased in the XXY group.

To investigate the effect of environmental factors and genotypes’ interactions on tobacco metabolites, we analysed all of the samples from two planting regions (XX and YJ), including three cultivars, with OPLS-DA. The result showed that six metabolites could be used as discriminative biomarkers in different regions, which were comp. **1**, comp. **2**, comp. **3**, comp. **5**, comp. **6**, and comp. **8**. Those of comp. **1**, comp. **2**, comp. **3**, comp. **5**, and comp. **6** were the same biomarkers from Yunyan 87 in YJ and XX (Figure 2C,D). This implied that the changes of these five metabolites were mainly correlated with environmental changes, regardless of their genotypes.

Although the environmental conditions demonstrated a greater effect, the genotype also played a major role in tobacco metabolism, which was verified by PCA. There were six compounds used as biomarkers to distinguish XXY and XXZ using OPLS-DA analysis (Figure 2E,F). Among them, comp. **4** and comp. **8** were also potential biomarkers of samples in different planting regions. Nevertheless, the other four metabolites were particularly discrepant substances between XXY and XXZ. 

### 3.3. Identification of Biomarkers

Structural identification was conducted by a UPLC-QTOF MS/MS analysis platform based on its retention time, accurate *m*/*z* and MS/MS data. We used a potential biomarker (comp. **3** molecular weight = 771.1967) to demonstrate the substance identification procedures. First, the accurate mass of the ion was obtained from UPLC Q-TOF/MS at ESI^−^ modes and the fragment ion was *m*/*z* 771.1967 ([M−H]^−^). The assistant software packed in the MassLynx i-FIT algorithm was used to determine the elemental composition for the peak at *m*/*z* 771.1967. The result showed that there were two candidates and that the i-FIT value of the first one (C_34_H_36_N_4_O_17_) was low, whereas the second one (C_33_H_40_O_21_) had a higher i-FIT value with a low tolerance. Second, some databases, such as KEGG, METLIN and Chemspider, were used to search for MS and MS^n^ information based on the accurate *m*/*z* and potential formula. Meanwhile, the MS/MS information (771.1967, 609.1401, 300.0269 and 271.0233) was obtained with collision energy from 10 to 60 V in the negative ion mode. The fragment ion at *m*/*z* 609.1401 was obtained through the loss of 163 (C_6_H_11_O_5_) from the ion at *m*/*z* 771.1967 ([M−H] ^−^). The fragment ion (*m*/*z* 609.1401, [M−H−C_6_H_11_O_5_]^−^) emerged at *m*/*z* 609.1401, 300.0269 and 271.0233 and were following fragments generated by rutin, which indicated that the structure of this fragment ion must include one rutin group and a hexose group. Therefore, the candidate from the database with neither a rutin group nor a hexose group was excluded. Finally, this candidate was tentatively identified as quercetin 3-rutinoside-7-glucoside, which has not previously been reported in tobacco but has been reported in tomato [13]. In addition, eleven potential biomarkers were identified by the methods described above, which were Malate, ouabagenin, taxezopidine K1, taxezopidine K2, taxezopidine K3 and some polyphenol metabolites (quinate, neochlorogenic acid, conjugated chlorogenic acid, rutin, chlorogenic acid, and quercetin 3-rutinoside-7-glucoside), whereas the other metabolite is still unknown (Table 2).

### 3.4. Influence of Genotype and Environmental Factor Interaction on the Accumulation of Tobacco Metabolites by Univariate Analysis

**Figure 3:**
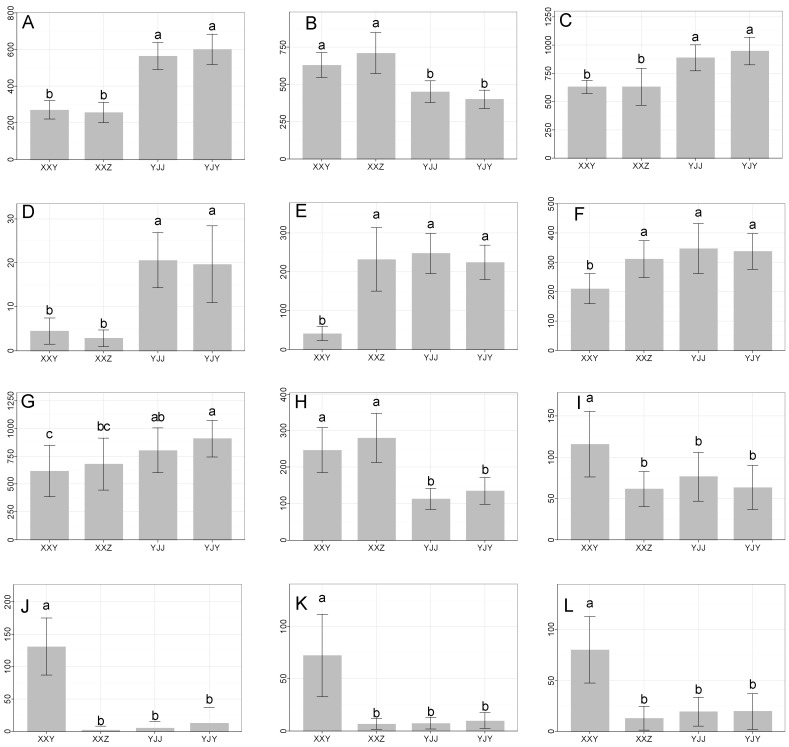
Bar plots of the biomarkers. From (**A**–**L**) represent the biomarkers (conjugated Chlorogenic acid, Malic acid, Chlorogenic acid, Quercetin 3-rutinoside-7-glucoside, quinate, neochlorogenic acid, Rutin, Unknown compound **5**, Ouabagenin, Taxezopidine K1, Taxezopidine K2, Taxezopidine K3 respectively); Different letters indicate significant differences at the level of *p* < 0.01 (Kruskal-Wallis ANOVA).

The relative contents of the biomarkers in different planting regions and different cultivars are shown in Figure 3. The K-W ANOVA showed that these biomarkers were statistically significant (*p* ≤ 0.01) in different regions or cultivars. Based on multiple comparisons, the metabolites malate, conjugated chlorogenic acid, chlorogenic acid, quercetin 3-rutinoside-7-glucoside, and unknown compound **5** were statistically significant in different planting regions. However, no differences were found between the two cultivars from the same planting region. This showed that environmental factors mainly influenced the accumulation of these five metabolites in tobacco. Malate is involved in various metabolic pathways [14] and implicated in several enzymes. One of the most important Malates metabolizing enzymes is the NADP-malic enzyme (NADP-ME), activated by UV-B radiation or wounding [15,16]. Since the intensity of UV-B radiation in Yanji (YJ) was greater than in Xiangxian (XX) in August, the activation of NADP-Me resulted in more Malate degradation. This explains why the content of Malate in XX was higher. Furthermore, this enzyme could provide NADPH for the biosynthesis of flavonoids, a pathway that requires significant reductive energy. Similarly, NADP-Me was linked to phosphoenolpyruvate (PEP) production, which was used in the shikimate pathway to produce aromatic amino acids such as phenylalanine. However, phenylalanine was the common precursor of polyphenol synthesis [15,16]. Polyphenols such as conjugated chlorogenic acid, chlorogenic acid, and quercetin 3-rutinoside-7-glucoside were found to be higher in YJ region, which was likely due to NADP-ME activity.

In our results, a significantly higher level of chlorogenic acid was observed in tobacco leaves from YJ, which had the longest sun exposure time and the lowest average temperature. The results suggest that chlorogenic acid increased with increasing latitudes, sun exposure time and decreasing temperature. Hårdh et al. [17] considered that the phenolic contents of plants were more pronounced in the northern latitudes compared with plants grown in the south. The latitudes could cause changes in the sun exposure hours and photoperiod. The sun exposure hours in the tobacco growth period in YJ (May–October) were 200 h longer than that in XX (Figure S1). Long day and cool night temperatures in the northern latitudes might be the reason for increased polyphenols [18].

Phenolics can accumulate in the vacuoles of plants as glycosides. Flavonols and flavones, particularly kaempferol and quercetin, were conjugated to sugars, mainly glucose, rhamnose and rutinose. Rutin is formed by the conjugate connection of quercetin and rutinose. The rutin content was significantly different between XXY and samples in YJ (YJY and YJJ). Likewise, YJY was different from samples in XX (XXY and XXZ). The content of rutin in different cultivars of the same planting regions had no significant difference. In addition, XXZ and YJJ had no significant difference. It is possible that rutin was affected by both environmental factors and cultivars, but that the environmental factors still played a major role.

Polyphenols were identified in this paper, and all of them were correlated with environmental changes. Coping with biotic and abiotic stress is the main biological function of polyphenols in plants [19], and the content of polyphenols reflects the mechanism of plant adaption to the environment [20]. The sensitivity of cultivars to the environment was different, which might be the reason why the content of polyphenols induced by environmental factors was discrepant in different cultivars. In this regard, we could screen cultivars adapting to the local environment based on the sensitivity of the cultivars.

## 4. Conclusions

The metabolites of tobacco leaves from different planting regions and cultivars were significantly different, and the influence of planting regions was greater than the cultivar influence. OPLS-DA selected twelve biomarkers, and seven of them were identified as polyphenols. Among these biomarkers, malate, conjugated chlorogenic acid, chlorogenic acid, quercetin 3-rutinoside-7-glucoside, and unknown compound **5** were only influenced by environmental factors independent of the genotype, whereas quinate, neochlorogenic acid, ouabagenin, taxezopidine K1, taxezopidine K2 and taxezopidine K3 in tobacco were influenced by the interaction of environmental factors and the genotype. The content of polyphenols reflects the mechanism of plant adaption to the environment. In agricultural practice, it is feasible to screen the cultivars that adapt better to the local environment according to the sensitivity of the cultivars and using the methods of metabolomics. The optimum mean daily temperature for growth is 20–30 °C; an atmospheric humidity of 80 to 85% and soil without high nitrogen levels is optimal. When grown commercially, tobacco requires a frost-free period of 90–120 days from transplanting to the last harvest of leaves. When grown as a crop, a dry period is required for the leaves to ripen and be harvested. Moreover, excessive rainfall causes thin, lightweight leaves. To develop its full aroma, sun-cured or oriental tobacco requires a relatively dry climate.

## Figures and Tables

**Table 1:** A list of sampling information on tobacco in China.

Growing Locations	Cultivars	Sampling Date
Xiangxian, Henan Province (XX)	Yunyan87 (XXY)	5 August
Xiangxian, Henan Province (XX)	Yunyan87 (XXY)	15 August
Xiangxian, Henan Province (XX)	Yunyan87 (XXY)	25 August
Xiangxian, Henan Province (XX)	Zhongyan100 (XXZ)	5 August
Xiangxian, Henan Province (XX)	Zhongyan100 (XXZ)	15 August
Xiangxian, Henan Province (XX)	Zhongyan100 (XXZ)	25 August
Yanji, Jilin Province (YJ)	Yunyan87 (YJY)	5 August
Yanji, Jilin Province (YJ)	Yunyan87 (YJY)	15 August
Yanji, Jilin Province (YJ)	Yunyan87 (YJY)	25 August
Yanji, Jilin Province (YJ)	Jiyan9 (YJJ)	5 August
Yanji, Jilin Province (YJ)	Jiyan9 (YJJ)	15 August
Yanji, Jilin Province (YJ)	Jiyan9 (YJJ)	25 August

**Table 2:** The biomarkers were identified by UPLC-QTOF MS/MS from the Tobacco Leaf.

Index	Retention Time (min)	Mass	Elemental Composition (ppm)	MS/MS	Compounds
compound **1**	4.35	707.181	0.2, C_32_H_36_O_18_	353, 191, 85	conjugated Chlorogenic acid
compound **2**	4.35	353.0873	0.0, C_16_H_17_O_9_	191, 85	Chlorogenic acid
compound **3**	5.18	771.1967	−0.4, C_33_H_40_O_21_	609, 301, 271	Quercetin 3-rutinoside-7-glucoside
compound **4**	7.15	609.1449	−1.1, C_27_H_29_O_16_	301, 271	Rutin (Quercetin 3-O-rutinoside)
compound **5**	0.59	377.0847	−0.3, C_22_H_17_O_4_S	341, 191	Unknown
compound **6**	0.62	133.0138	0.8, C_4_H_5_O_5_	115, 71	Malic acid
compound **7**	12.51	437.2174	−0.2, C_23_H_34_O_8_	395, 377, 233, 116	Ouabagenin
compound **8**	4.8	353.0871	−0.6, C_16_H_17_O_9_	191, 173, 135	neochlorogenic acid
compound **9**	0.6	191.0555	−0.5, C_7_H_11_O_6_	85	quinate
compound **10**	16.81	681.2962	−1.2, C_37_H_46_O_12_	292, 116	Taxezopidine K1
compound **11**	17.04	681.2962	−1.2, C_37_H_46_O_12_	292, 146, 116	Taxezopidine K2
compound **l2**	16.55	681.2957	−1.2, C_37_H_46_O_12_	146, 116	Taxezopidine K3

## Supplementary Materials

The following supporting information can be downloaded at: https://www.mdpi.com/article/10.3390/life12040590/s1, Figure S1: Shows the recorded meteorological conditions for Yanji and Xiangxian regions.
