# Supplementary material for: Influence of Genotypic and Environmental Factors on Tobacco Leaves Based on Metabolomics
Source: Life (Basel). 2022 Apr 15;12(4):590. doi: 10.3390/life12040590 (PMC9025834; doi:10.3390/life12040590)
Supplement: Supplementary file 1 [file life-12-00590-s001.zip › life-1663505-supplementary.pdf]

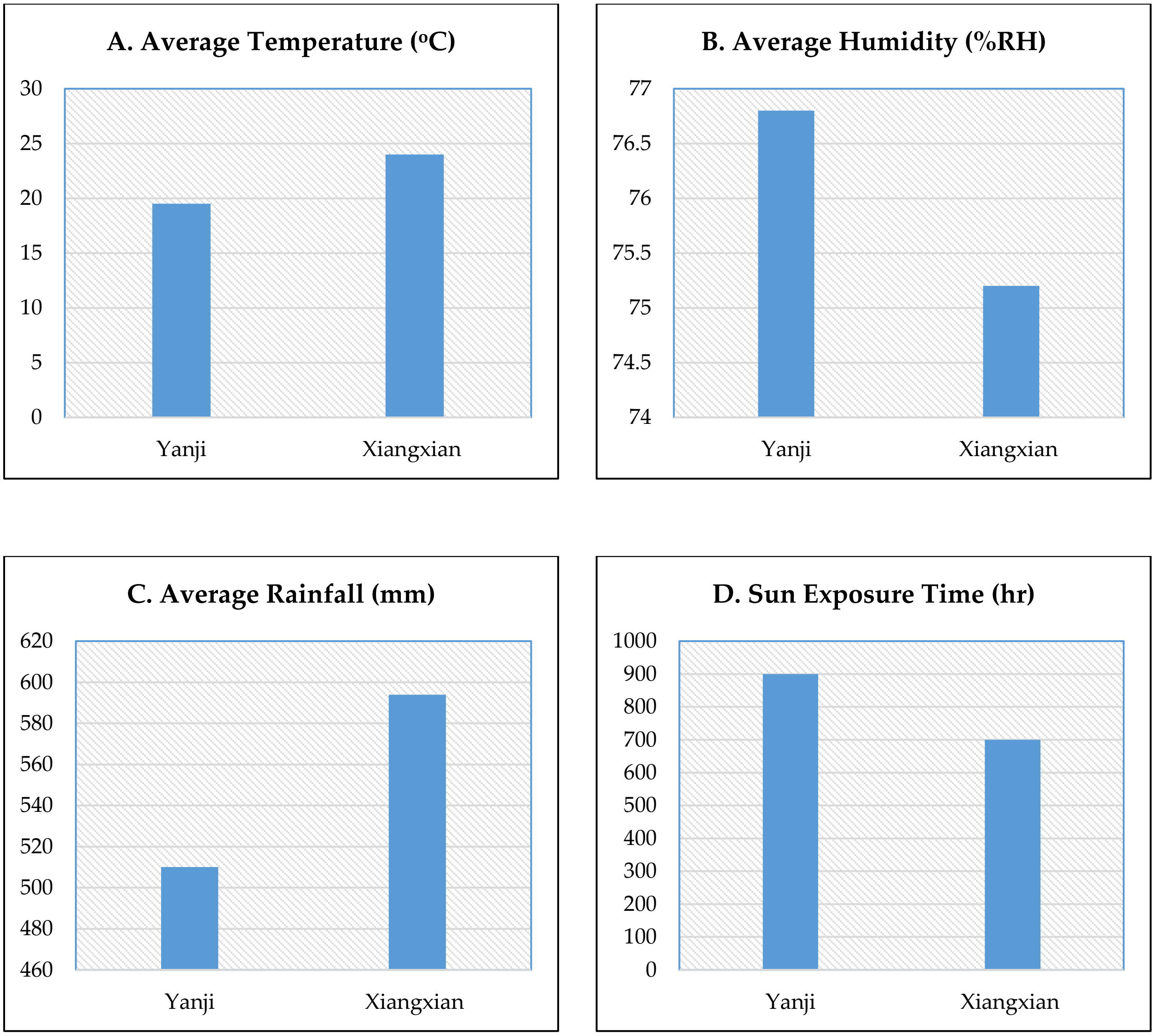

Figure S1. Shows the recorded meteorological conditions for Yanji and Xiangxian regions: A. Average temperature (°C); B. Average humidity (%RH); C. Average rainfall (mm); and D. Sun exposure time (hr), during current study (from May to October, 2015).
